# Genome Sequence of the Electrogenic Petroleum-Degrading *Thalassospira* sp. Strain HJ

**DOI:** 10.1128/genomeA.00483-15

**Published:** 2015-05-14

**Authors:** Larisa Kiseleva, Sofya K. Garushyants, Justina Briliute, David J. W. Simpson, Michael F. Cohen, Igor Goryanin

**Affiliations:** a Biological Systems Unit, Okinawa Institute of Science and Technology, Tancha, Onna-son, Okinawa, Japan; b A. A. Kharkevich Institute for Information Transmission Problems, Moscow, Russia; c School of Informatics, University of Edinburgh, Edinburgh, United Kingdom

## Abstract

We present the draft genome of the petroleum-degrading *Thalassospira* sp. strain HJ, isolated from tidal marine sediment. Knowledge of this genomic information will inform studies on electrogenesis and means to degrade environmental organic contaminants, including compounds found in petroleum.

## GENOME ANNOUNCEMENT

Inadvertently, through oil spills large and small, humans have amplified petroleum-degrading microorganisms on an unprecedented global scale (1). Strains isolated from shoreline sediments that regularly receive petroleum contaminants could potentially be exploited to break down petroleum in controlled environments, such as in microbial fuel cells (2).

Members of genus *Thalassospira* are Gram-negative, halophilic, and often facultative anaerobic alphaproteobacteria with the ability to utilize hydrocarbons as their sole source of carbon and energy (3). There are currently eight validly named species of *Thalassospira* (http://www.bacterio.net), and the genomes of seven strains are available in the GenBank database (https://www.ncbi.nlm.nih.gov/genbank/). Strain HJ was isolated from stagnant tidal marine sediment at Kaichu-doro Beach, Okinawa, Japan (26°19′56.1″N 127°54′0″E), in October 2013, 5 km from a large petroleum storage facility that has been in operation since the early 1970s. Strain HJ can grow on petroleum as a sole carbon source, it generates power in a microbial fuel cell and, based on *in silico* studies, and it possesses several catabolic pathways of interest (our unpublished data).

The genome of strain HJ was sequenced using the Illumina MiSeq sequencing platform (Illumina, Inc., San Diego, CA). The generated reads were trimmed and assembled *de novo* using SPAdes version 3.1.1 (4). The resulting sequence was then submitted to the NCBI Prokaryotic Genomes Automatic Annotation Pipeline for autoannotation (5). The size of the genome of strain HJ was found to be 4,269,399 bp, comprising 22 contigs, with a G+C content of 54.9%. Strain HJ contains 3,909 predicted genes, 3,838 putative coding sequences (CDS), 7 rRNAs, and 57 tRNAs.

*In silico* DNA-DNA hybridization (DDH) comparisons (6) between the genome of strain HJ and other accessible *Thalassospira* genome sequences in GenBank indicate the closest match to *Thalassospira profundimaris* WP0211 (7), with an estimated DDH value of 73.2% ± 3.8%, while comparisons with other *Thalassospira* strains gave values from 12.8% to 57.0% (high-scoring segment pair length/total length, generalized linear model based). The aligned 16S rRNA gene sequences of these two organisms show 99.7% identity (1,449 bp/1,453 bp).

### Nucleotide sequence accession number. 

The genome data have been deposited at NCBI under BioProject no. PRJNA275638 and accession no. JYII00000000 for *Thalassospira* sp. HJ.

## References

[1] Head IM, Jones DM, Röling WF 2006 Marine microorganisms make a meal of oil. Nat Rev Microbiol 4:173–182. doi:10.1038/nrmicro1348. 16489346

[2] Wang X, Cai Z, Zhou Q, Zhang Z, Chen C 2012 Bioelectrochemical stimulation of petroleum hydrocarbon degradation in saline soil using U-tube microbial fuel cells. Biotechnol Bioeng 109:426–433. doi:10.1002/bit.23351. 22006588

[3] Baldani JI, Videira SS, dos Santos Teixeira KR, Reis VM, de Oliveira ALM, Schwab S, de Souza EM, Pedraza RO, Baldani VLD, Hartmann A 2014 The family *Rhodospirillaceae*, p 533–618. *In* Rosenberg E, DeLong EF, Lory S, Stackebrandt E, Thompson F (ed), The prokaryotes. Springer Verlag, Heidelberg, Germany.

[4] Nurk S, Bankevich A, Antipov D, Gurevich A, Korobeynikov A, Lapidus A, Prjibelsky A, Pyshkin A, Sirotkin A, Sirotkin Y, Stepanauskas R, McLean J, Lasken R, Clingenpeel SR, Woyke T, Tesler G, Alekseyev MA, Pevzner PA 2013 Assembling genomes and mini-metagenomes from highly chimeric reads, p 158–170. *In* Deng M, Jiang R, Sun F, Zhang X (ed), Research in computational molecular biology. Springer Verlag, Heidelberg, Germany. 10.1089/cmb.2013.0084 PMC3791033 24093227

[5] Tatusova T, DiCuccio M, Badretdin A, Chetvernin V, Ciufo S, Li W 2013 Prokaryotic genome annotation pipeline. The NCBI handbook [Internet], 2nd ed. National Center for Biotechnology Information, Bethesda, MD.

[6] Meier-Kolthoff JP, Auch AF, Klenk H-P, Göker M 2013 Genome sequence-based species delimitation with confidence intervals and improved distance functions. BMC Bioinformatics 14:60. doi:10.1186/1471-2105-14-60. 23432962 PMC3665452

[7] Lai Q, Shao Z 2012 Genome sequence of *Thalassospira profundimaris* type strain WP0211. J Bacteriol 194:6957. doi:10.1128/JB.01903-12. 23209215 PMC3510614

